# Association of T cell infiltration and morphological change of thymus gland with the aggravation of pulmonary emphysema in testosterone deficiency

**DOI:** 10.1016/j.bbrep.2023.101489

**Published:** 2023-05-25

**Authors:** Kentaro Aono, Junichi Matsumoto, Taichi Matsumoto, Mitsuhisa Koga, Keisuke Migita, Koji Tominaga, Shinsuke Nakagawa, Atsushi Yamauchi

**Affiliations:** a Department of Pharmaceutical Care and Health Sciences, Faculty of Pharmaceutical Sciences, Fukuoka University, 8-19-1 Nanakuma, Jonan-ku, Fukuoka, 814-0180, Japan; b Department of Drug Informatics, Faculty of Pharmaceutical Sciences, Fukuoka University, 8-19-1 Nanakuma, Jonan-ku, Fukuoka, 814-0180, Japan; c Laboratory of Drug Design and Drug Delivery, Faculty of Pharmaceutical Sciences, Fukuoka University, 8-19-1 Nanakuma, Jonan-ku, Fukuoka, 814-0180, Japan; d Department of Pharmaceutical and Health Care Management, Faculty of Pharmaceutical Sciences, Fukuoka University, 8-19-1 Nanakuma, Jonan-ku, Fukuoka, 814-0180, Japan

**Keywords:** Chronic obstructive pulmonary disease, Testosterone, Emphysema, T cell, Thymus gland

## Abstract

Chronic obstructive pulmonary disease is an inflammatory lung disease characterized by chronic bronchitis and emphysema. Our previous study revealed that testosterone depletion induced T cell infiltration in the lungs and aggravated pulmonary emphysema in orchiectomized (ORX) mice exposed to porcine pancreatic elastase (PPE). However, the association between T cell infiltration and emphysema remains unclear. The aim of this study was to determine whether thymus and T cells are involved in the exacerbation of PPE-induced emphysema in ORX mice. The weight of thymus gland in ORX mice was significantly greater than that of sham mice. The pretreatment of anti-CD3 antibody suppressed PPE-induced thymic enlargement and T cell infiltration in the lungs in ORX mice, resulting in improved expansion of the alveolar diameter, a marker of emphysema exacerbation. These results suggest that increased thymic function due to testosterone deficiency and the associated increased pulmonary infiltration of T cells may trigger the development of emphysema.

## Highlights

• Orchiectomized mice showed thymic enlargement and T-cell lung infiltration.
• Suppression of thymic hyperplasia and T-cell infiltration prevented emphysema.
• Thymus and T cells play an important role in testosterone deficiency emphysema.

## 1 Introduction

Chronic obstructive pulmonary disease (COPD) is a global health problem affecting approximately 210 million people and is the third leading cause of death worldwide [1]. COPD is characterized by progressive airflow obstruction caused by peripheral airway obstruction and emphysema due to inflammation induced by inhaling harmful particles from smoking and air pollution [2,3]. Epidemiological studies have identified intrinsic factors that contribute to these pathological changes, such as genetic alterations, airway sensitivity, autoimmunity, aging, and sex-related differences [4,5]. Recent cross-sectional studies on COPD and sex-related differences revealed that emphysema occurs more in men than women, regardless of their smoking status (non-smokers, ex-smokers, or smokers) [6]. However, the mechanisms underlying the differences in emphysema development between men and women have yet to be fully investigated.

The immune system plays a crucial role in the development of COPD. Neutrophils and macrophages are traditionally thought to be central to emphysema development, as they produce proteases that contribute to the breakdown of lung tissue [7,8]. Recent research has focused on the role of other immune cells, such as eosinophils, basophils, and T cells, in COPD pathogenesis [9,10]. Accumulation of T cells, particularly helper and cytotoxic T cells, has been observed in the lungs of COPD patients, and they exacerbate inflammation through proinflammatory cytokines production [11,12]. Therefore, T cell accumulation may contribute to the persistent inflammation characteristic of COPD [13].

A meta-analysis demonstrated that COPD patients have significantly lower serum testosterone levels than healthy controls [14]. Sex hormones influence immune function in men and women [15,16], with testosterone functioning as an inhibitory agent via androgen receptors on various immune cells, including T cells [17,18]. Furthermore, testosterone promotes T cell development and maturation through apoptosis induction on the thymus gland, a T cell-producing organ [19]. These findings suggest that testosterone deficiency may contribute to COPD progression by affecting T cell immunity.

We recently reported that testosterone deficiency exacerbates emphysema in a porcine pancreatic elastase (PPE)-induced mouse model and that the lung infiltration of mature T cells, such as CD3^+^CD4^+^ cell (helper T cells) and CD3^+^CD8^+^ cells (killer T cells), is increased in these mice [20]. The PPE-induced emphysema model is considered to be an acute phase model that recapitulates the protease-antiprotease imbalance in COPD pathogenesis [9,21]. Therefore, we hypothesized that increased T-cell pulmonary infiltration caused by testosterone deficiency induces acute inflammation and contributes to emphysema pathogenesis. In this study, to clarify this hypothesis, we investigated the effects of depletion of mature T cells using CD3 antibodies on PPE-induced emphysema development. We also examined the role of the thymus, the organ of T cell differentiation and maturation.

## 2 Methods

### 2.1 Animals and experimental design

All animal experiments adhered to the Law for the Protection and Management of Animals (Law No. 105), and the Standards Relating to the Care and Management of Experimental Animals (Notification No. 6) of the Japanese Government and were approved by the Laboratory Animal Care and Use Committee of Fukuoka University (No. 2206021, June 29, 2022). Seven-week-old male C57BL/6J mice were purchased from KBT Oriental Co. (Saga, Japan). The mice were provided food and water ad libitum. All animals were housed in a specific pathogen-free and 12:12 h light-dark cycle environment. Mice were acclimatized to the rearing environment for one week and randomly distributed into two experimental groups: the sham and orchiectomized (ORX) groups. Mice in the ORX group were anesthetized using an intraperitoneal injection of medetomidine (0.3 mg/kg), midazolam (4 mg/kg), and butorphanol tartrate (5 mg/kg), and the testicles were surgically removed from the lower abdomen. The sham group underwent the same procedure but without orchiectomy. Two weeks after surgery, the mice were assigned to one of four experimental subgroups: the sham (n = 8), ORX (n = 7), sham/PPE (n = 18), and ORX/PPE groups (n = 16). Intrabronchial administration of PPE was performed according to previous study [20]. Briefly, the mice in the PPE groups were anesthetized by subjecting them to isoflurane inhalation and intrabronchial administration of 0.5 units PPE (Sigma-Aldrich, St. Louis, MO, USA) in sterile phosphate buffered saline (PBS) with a MicroSprayer Aerosolizer drug delivery device (Penn-Century Inc., Wyndmoor, PA, USA).

### 2.2 Thymus histology

Fourteen days after orchiectomy, mice assigned for alveolar lavage fluid collection were anesthetized, and their thymus was harvested. Thymic tissue staining was performed according to previous study [22]. The thymus was isolated from the mice and weighed. The two lobes of each thymus were physically separated, and the left thymus was fixed by immersion in 4% paraformaldehyde (PFA) at 4 °C for 1 d. Thymus sections (10 μm thick) were prepared and stained with hematoxylin and eosin (H&E) after dehydration using 30% sucrose. Three different sections were captured for each mouse with an all-in-one fluorescence microscope (BZ-X710, KEYENCE, Osaka, Japan) using a Plan Apochromat 2x objective (NA0.10, BZ-PA02, KEYENCE, Osaka, Japan), and each section was analyzed using the BZ-X analyzer software (Keyence Corporation, Osaka, Japan).

### 2.3 Bronchoalveolar lavage fluid

Mice designated for flow cytometry were anesthetized, and bronchoalveolar lavage fluid (BALF) was obtained as previously described [20]. BALF samples were centrifuged, and the cell pellet was resuspended in a fluorescence-activated cell sorting (FACS) buffer.

### 2.4 Flow cytometry

BALF cell suspensions were stained with fluorescent specific antibodies (Table 1) as previously described [20]. Fluorescence emissions were assessed by flow cytometry using BD FACSVerse Flow Cytometer (BD Biosciences, San Jose, CA, USA) and FlowJo software (TreeStar, Ashland, OR, USA). The gating strategy is shown in Supplemental Fig. S1.

**Table 1:** List of fluorescent specific antibodies used.

Marker	Fluorochrome	Clone	Dilution	Provider	Cell Target
7-AAD			1:100	Biolegend	Dead cells
CD45	BV421	30-F11	1:300	Biolegend	Leukocytes
CD3	FITC	145-2C11	1:300	Biolegend	T lymphocytes
		17A2 *^1^			
CD4	PE-Cy7	GK1.5	1:300	Biolegend	Th lymphocytes
CD8	BV510	53–6.7	1:100	Biolegend	Tc lymphocytes

*^1^ Used in experiments with anti-CD3 antibody administration. Th, T helper; Tc, T cytotoxic.

### 2.5 Administration of anti-CD3 antibody

The administration of the anti-CD3 antibody was performed by the method used in the previous studies with minor modifications [[22], [23], [24]]. In brief, the anti-CD3 antibody (Clone: 145-2C11, Thermo Fisher Scientific, Waltham, MA, USA) was diluted 10-fold with water for the injection. The sham/PPE and ORX/PPE mice were intraperitoneally administered anti-CD3 antibody (10 μg/mouse/day) or isotype control mAb (Thermo Fisher Scientific, Waltham, MA, USA). Antibodies were administered on three consecutive days prior to PPE treatment and on day 4 after PPE administration.

### 2.6 Lung histology

Three weeks post-PPE inhalation, the mice were anesthetized. The left lung was obtained and fixed by immersion in 4% PFA at 4 °C for 2 d. After dehydration using 30% sucrose, the lung was embedded in an optimal cutting temperature (OCT) compound (Sakura Finetek, Tokyo, Japan) and sliced into 7 μm thick sections. Lung sections were stained using H&E. Histopathological images were obtained using a BZ-X710 microscope using a Plan Apochromat 20x objective (NA0.75, BZ-PA20, KEYENCE, Osaka, Japan), As an indicator of lung pathology, the mean linear intercept (MLI), which is the amount of airspace size between the alveolar walls, was calculated using Adaptive Vision Studio 4.9 Lite software (Future Processing, Gliwice, Poland) as previously described [25]. Five randomly selected microscopic fields per section, of three different sections (15 fields), were observed and analyzed for each mouse.

### 2.7 Statistical analysis

GraphPad Prism 5.0 (GraphPad, San Diego, CA, USA) was used for all statistical analyses. Data are presented as the mean ± standard error of the mean (SEM). The statistical significance of differences between two groups was assessed using the Student's t-test and one-way analysis of variance (ANOVA) with Tukey-Kramer's post-hoc test for multiple comparisons. Differences of p-values < 0.05 were considered statistically significant.

## 3 Result

### 3.1 Orchiectomy enlarged the thymus and increased the number of T cells in BALF

**Fig. 1:**
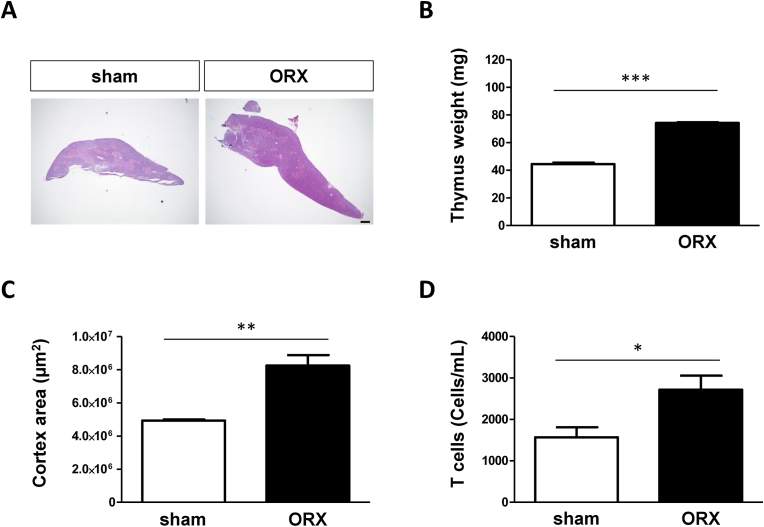
*Orchiectomy enlarged the thymus and increased the number of T cells in the Lung* (A) Representative images of hematoxylin and eosin (H&E)-stained thymus sections. Scale bar = 400 μm, (B) thymic weight, and (C) thymic cortex area from the sham and orchiectomized (ORX) mice two weeks after surgery. (D) The number of T cells in the bronchoalveolar lavage fluid (BALF) was obtained from the sham and ORX groups. Each bar represents the mean ± standard error of the mean (SEM); n ≥ 4 mice in each group; *p < 0.05, **p < 0.01, ***p < 0.001 by unpaired *t*-test.

To evaluate the effects of orchiectomy on the lungs and thymus two weeks post-surgery, thymus morphology and lung T cell counts in ORX mice were analyzed. ORX mice had enlarged thymus, and thymic weight and cortex area were significantly increased compared with that in sham mice (Fig. 1A–C). Moreover, a significantly larger number of infiltrated T cells were observed in the BALF of ORX mice than of sham mice (Fig. 1D).

### 3.2 Anti-CD3 antibody administration attenuated alveolar destruction in ORX mice with PPE-induced emphysema

**Fig. 2:**
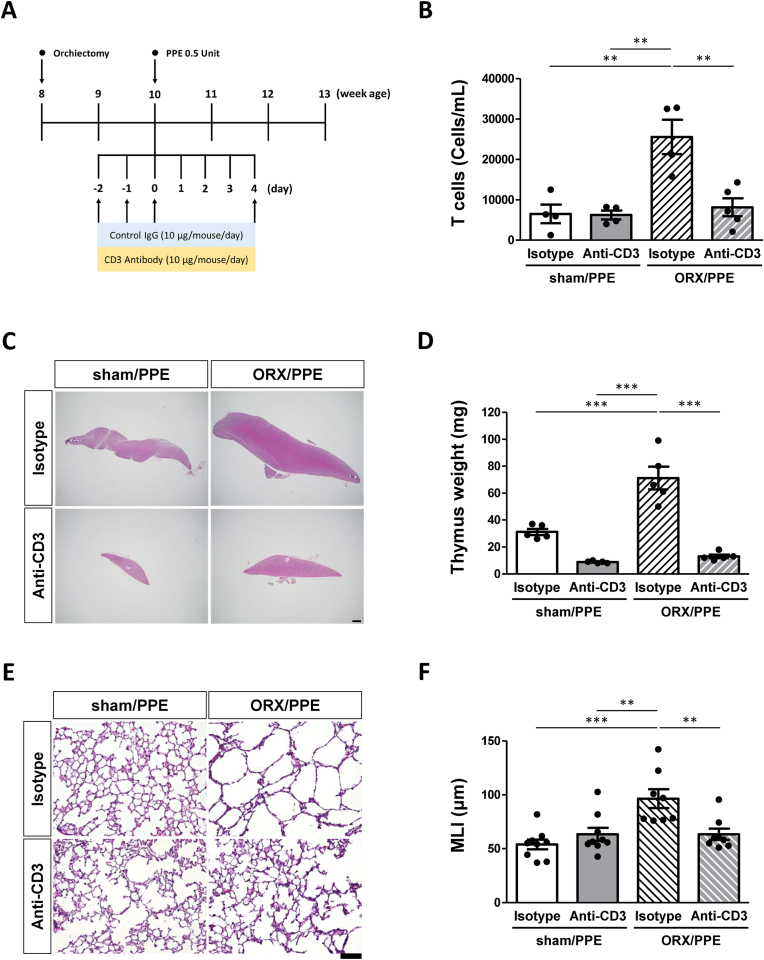
*Effect of anti-CD3 antibody treatment on thymus and pulmonary pathological change in ORX/PPE mice*. (A) Schematic representation of the experimental design for ORX surgery, and anti-CD3 antibody administration and porcine pancreatic elastase (PPE) inhalation. (B) The number of T cells in BALF obtained from each group treated with isotype or anti-CD3 two days after PBS or PPE exposure. The BALF was analyzed using flow cytometry. n ≥ 4 mice in each group. (C) Representative photomicrographs of H&E-stained thymus sections obtained from each mice group two days after PBS or PPE exposure. Scale bar = 400 μm. (D) Thymic weight in each group two days after PPE exposure. n ≥ 5 mice in each group. (E) Representative photomicrographs of H&E-stained lung sections obtained from each mice group 21 days after PBS or PPE exposure. Scale bar = 100 μm. (F) mean linear intercept (MLI) quantification for each mice group 21 days after PPE inhalation. n ≥ 8 mice in each group. Each bar represents the mean + SEM, **p < 0.01, ***p < 0.001 by one-way analysis of variance (ANOVA).

We next examined the effect of T cell removal on emphysema formation by pretreatment with anti-CD3 antibodies. T cell counts in BALF and thymus weights were measured two days after PPE administration. The number of T cells in ORX/anti-CD3 mice was significantly lower than that in ORX/isotype mice (Fig. 2B). Anti-CD3 antibody treatment reduced the apparent size of the thymus gland and its weight was significantly reduced compared to isotype-treated mice (Fig. 2C and D). The alveoli of ORX/isotype mice 21 days after PPE administration were enlarged compared with the sham mice, and this enlargement was suppressed in ORX/anti-CD3 mice (Fig. 2E). The quantitative analysis by calculating MLI showed that increased MLI in ORX/isotype mice was significantly suppressed in ORX/anti-CD3 mice (Fig. 2F).

## 4 Discussion

Recent cross-sectional studies have demonstrated an inverse relationship between COPD severity and serum testosterone level [26]. In addition, COPD severity has been correlated with lung T cell accumulation [12,13]. These studies suggest that decreased testosterone levels may contribute to lung T cell accumulation and the pathological progression of COPD. In this study, in ORX mice, thymic enlargement and increased pulmonary T-cell accumulation were observed prior to PPE administration. However, these were not observed in ORX mice treated with anti-CD3 antibody, and emphysema formation was suppressed. These results suggest that testosterone deficiency-induced thymic tissue hypertrophy and increased T cell infiltration may trigger emphysema development.

Testes produce over 90% of testosterone in men [27], and its removal significantly reduces serum testosterone levels. Testosterone affects thymic morphology and function via androgen receptors [23,24]. The thymus is the organ of differentiation and maturation of T cells. Our previous study has also shown that serum testosterone levels are significantly reduced in ORX mice (Supplemental Fig. S2). In the present study, the thymic weight and cortical area in ORX mice increased significantly compared with that in the sham mice (Fig. 1B and C). Additionally, the number of T cells in the BALF was significantly increased (Fig. 1D). These results suggest that the lungs of ORX mice may be more susceptible to inflammation due to testosterone deficiency.

In a previous study, anti-CD3 antibody administration decreased thymus weight and removed peripheral T cells [28]. Therefore, to investigate the effects of ORX-induced thymus enlargement and T cell increase on emphysema development, anti-CD3 antibody-treated mice were subjected to PPE inhalation to induce emphysema. The number of T cells in the lung tissue and thymus weight of ORX/anti-CD3 antibody-treated mice were significantly lower than those of isotype-treated mice (Fig. 2B–D). In sham mice, no difference in T cell counts was observed between anti-CD3 antibody- and isotype-treated mice. The lung histology by H&E staining on day 21 after PPE administration revealed that emphysema formation was suppressed in ORX/anti-CD3 antibody-treated mice compared to that in isotype-treated mice (Fig. 2E and F). MLI is an indicator of emphysema progression and has been reported to be related to lung function [[29], [30], [31], [32]]. Thus, T cell depletion by CD3 antibody suppressed emphysema development in ORX mice. Gary et al. [33] reported that thymic enlargement associated with testosterone deficiency increased T cells in the periphery, and that the increased T cells in pulmonary inflammation were mainly due to migration from the periphery. In addition, mesenchymal cell administration suppressed thymic enlargement and emphysema induced by intermittent PPE administration [34]. These results suggest that increased T cells in BALF during the acute inflammatory phase may be directly correlated with pulmonary emphysema development in ORX mice. In our experiments, the CD3 antibody was used to decrease overall mature T cells. Further studies are needed to determine which T cell subtypes contribute to emphysema formation.

In conclusion, ORX causes testosterone deficiency and thymic enlargement, and may increase lung T cell accumulation and contribute to emphysema development. Therefore, under conditions of testosterone depletion, such as in older men, the thymus and T cells may play an important role in COPD pathogenesis.

## Author contributions

**Kentaro Aono**: Conceptualization, Investigation, Writing - Original Draft, Funding acquisition, **Junichi Matsumoto**: Conceptualization, Investigation, Funding acquisition, **Taichi Matsumoto**: Investigation, Resources, **Mitsuhisa Koga**: Investigation, Resources, **Keisuke Migita**: Supervision, Resources, **Koji Tominaga**: Supervision, Writing - Review & Editing, **Shinsuke Nakagawa**: Supervision, Writing - Review & Editing, **Atsushi Yamauchi**: Conceptualization, Project administration, Funding acquisition, Writing - Review & Editing.

## Funding

This work was supported in part by the 10.13039/501100001691Japan Society for the Promotion of Science (grant number 22K20729 to K.A., 22K06715 to J.M. and 21K06683 to A.Y.) and 10.13039/501100004330Smoking Research Foundation (grant number 2022Y008 to J.M.).

## Declaration of competing interest

The authors declare that they have no known competing financial interests or personal relationships that could have appeared to influence the work reported in this paper.

## Appendix A Supplementary data

Supplemental figure S1. Representative images of the flow cytometry gating strategy for the identification of T cells, CD4^+^ T cells, and CD8^+^ T cells.

Supplemental figure S2. Serum testosterone concentration on 2 weeks after ORX or sham operations. Each bar represents the mean + S.E.M. ***p < 0.01 by unpaired *t*-test.
