# Supplementary material for: Association of T cell infiltration and morphological change of thymus gland with the aggravation of pulmonary emphysema in testosterone deficiency
Source: Biochem Biophys Rep. 2023 May 25;34:101489. doi: 10.1016/j.bbrep.2023.101489 (PMC10220313; doi:10.1016/j.bbrep.2023.101489)
Supplement: Multimedia component 1 [file mmc1.pptx]

## Slide 1
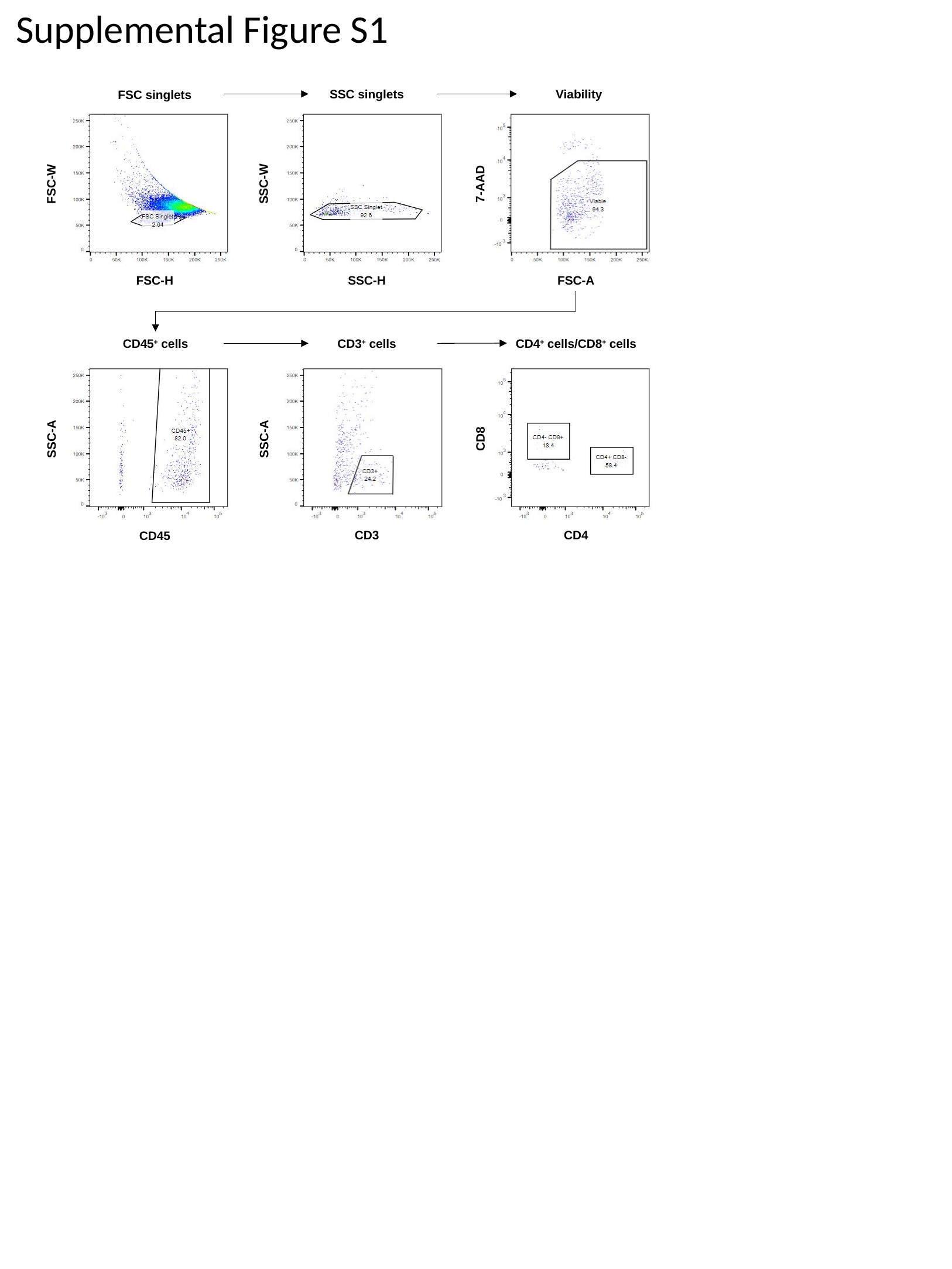

Supplemental Figure S1
SSC singlets
Viability
FSC singlets
FSC-W
7-AAD
SSC-W
FSC-A
SSC-H
FSC-H
CD45+ cells
CD3+ cells
CD4+ cells/CD8+ cells
SSC-A
CD8
SSC-A
CD4
CD3
CD45

## Slide 2
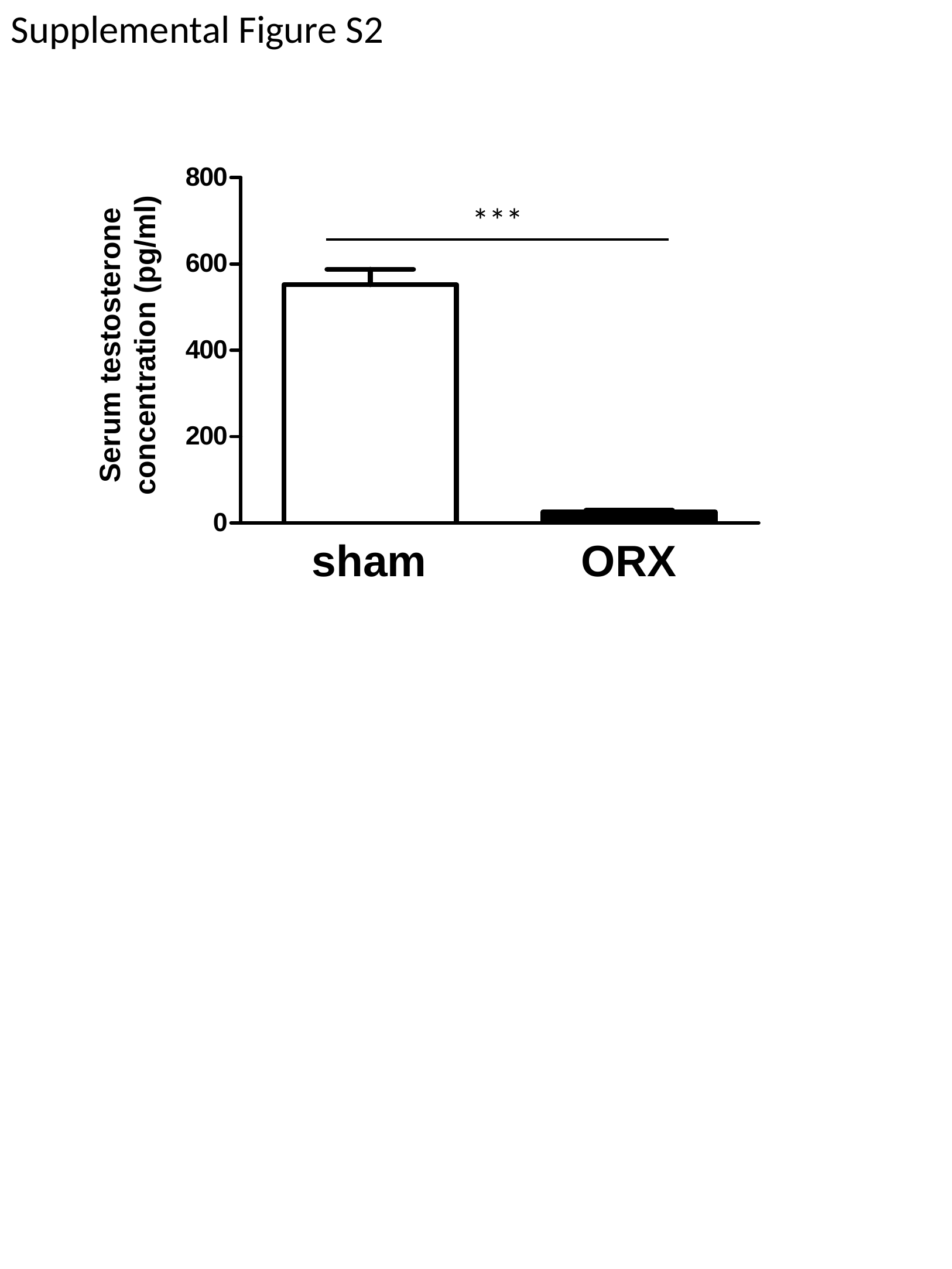

Supplemental Figure S2
***
Serum testosterone
concentration (pg/ml)
sham
ORX
